# Supplementation with Guanidinoacetic Acid in Women with Chronic Fatigue Syndrome

**DOI:** 10.3390/nu8020072

**Published:** 2016-01-29

**Authors:** Sergej M. Ostojic, Marko Stojanovic, Patrik Drid, Jay R. Hoffman, Damir Sekulic, Natasa Zenic

**Affiliations:** 1 Faculty of Sport and Physical Education, University of Novi Sad, Novi Sad 21000, Serbia; marko.stojanovic@chess.edu.rs (M.S.); patrikdrid@gmail.com (P.D.); 2 School of Medicine, University of Belgrade, Belgrade 11000, Serbia; 3 Department of Educational and Human Sciences, Burnett School of Biomedical Sciences, University of Central Florida, Orlando 32816, FL, USA; jay.hoffman@ucf.edu; 4 Faculty of Kinesiology, University of Split, Split 21000, Croatia; dado@kifst.hr (D.S.); natasa@kifst.hr (N.Z.); 5 Department of Health Care Studies, University of Split, Split 21000, Croatia

**Keywords:** cellular bioenergetics, creatine, general fatigue, exercise performance

## Abstract

A variety of dietary interventions has been used in the management of chronic fatigue syndrome (CFS), yet no therapeutic modality has demonstrated conclusive positive results in terms of effectiveness. The main aim of this study was to evaluate the effects of orally administered guanidinoacetic acid (GAA) on multidimensional fatigue inventory (MFI), musculoskeletal soreness, health-related quality of life, exercise performance, screening laboratory studies, and the occurrence of adverse events in women with CFS. Twenty-one women (age 39.3 ± 8.8 years, weight 62.8 ± 8.5 kg, height 169.5 ± 5.8 cm) who fulfilled the 1994 Centers for Disease Control and Prevention criteria for CFS were randomized in a double-blind, cross-over design, from 1 September 2014 through 31 May 2015, to receive either GAA (2.4 grams per day) or placebo (cellulose) by oral administration for three months, with a two-month wash-out period. No effects of intervention were found for the primary efficacy outcome (MFI score for general fatigue), and musculoskeletal pain at rest and during activity. After three months of intervention, participants receiving GAA significantly increased muscular creatine levels compared with the placebo group (36.3% *vs.* 2.4%; *p* < 0.01). Furthermore, changes from baseline in muscular strength and aerobic power were significantly greater in the GAA group compared with placebo (*p* < 0.05). Results from this study indicated that supplemental GAA can positively affect creatine metabolism and work capacity in women with CFS, yet GAA had no effect on main clinical outcomes, such as general fatigue and musculoskeletal soreness.

## 1. Introduction

Chronic fatigue syndrome (CFS) is a debilitating and complex medical condition characterized by profound fatigue of unknown cause, which is permanent and limits the patient’s functional capacity, producing various degrees of disability [1]. Given its unknown etiology, different hypotheses have been considered to explain the origin of this perplexing condition [2]. It seems that inadequate or impaired energy provision through cellular metabolism may contribute to the pathogenic initiation and maintenance of CFS [3]. CFS occurs with a wide spectrum of signs and symptoms, ranging from fatigue, general muscular pain and weakness through migratory arthralgias, painful axillar or cervical lymphadenopathy, neurocognitive and sleep disorders [4]. CFS represents a major healthcare problem, with the latest epidemiological studies showing prevalence rates as high as 3.3% of the general population [5], and it is found to be most prevalent in adults [5,6,7]. Individuals with CFS are reported to accumulate mean annual medical costs of up to $6000 in the US [8], while CFS-related annual global loss of productivity is approximately $7 billion [1]. High healthcare and socioeconomic burdens of CFS emphasize the need for the development of an effective and applicable therapeutic approach.

A variety of dietary interventions has been used in the management of CFS, yet no therapeutic modality has demonstrated conclusive positive results in terms of effectiveness [9]. Previous studies have evaluated the effects of essential fatty acids, vitamins, minerals and/or enzymes; findings do not support the use of a broad-spectrum nutritional supplement in treating CFS-related symptoms [10,11]. Considering that patients with CFS have lower levels of high-energy compounds (e.g., phosphocreatine, adenosine triphosphate) [3], an effective dietary treatment of CFS should be focused on providing compounds that facilitate cellular bioenergetics. Guanidinoacetic acid (GAA) could be of particular interest since it occurs naturally in the human body and acts as an immediate precursor of creatine [12]. Due to its low cost and high bioavailability [13], if proven effective dietary GAA may be suitable for use in a broad CFS population. Early clinical studies from the 1950’s revealed favorable effects of GAA (also known as glycocyamine) in patients with chronic illness, including heart disease, arthritis, and depression [14]. Overall, treatment with GAA was shown to lead to an improved sense of well-being and less fatigue, yet these studies did not appear to examine changes in cellular bioenergetics after GAA administration. Furthermore, only limited information was reported about the effect of GAA on clinical markers of patients’ health status. In a recent investigation, supplemental GAA was applied for several weeks in healthy humans [15]. Intervention caused a significant increase in fasting serum creatine concentrations (up to 50% after six weeks), with GAA demonstrating a low incidence of biochemical and clinical abnormalities. This implies that GAA supplementation in healthy humans may elevate the body’s own creatine pool, yet its clinical relevance is not clear. Preliminary evidence from our group in CFS patients revealed beneficial effects of four weeks of oral GAA application on myalgia and muscular strength in five young adults (age 18–21 years) suffering from this clinical condition (Ostojic *et al.*, unpublished data). However, to the best of our knowledge there is no evidence demonstrating that GAA administration increases intramuscular concentrations of creatine. There also appears to be a need for research on evaluating GAA clinical efficacy, metabolic behavior and safety during prolonged treatment periods (>one month) in adult CFS patients. Thus, the purpose of this study was to determine the effect of three months of GAA supplementation on CFS symptomatology using a fatigue severity inventory, soreness of locomotive apparatus scales, and a health-related quality of life survey. A secondary purpose was to determine the effect of GAA ingestion on creatine metabolism and exercise performance.

## 2. Methods

### 2.1. Study Population

A placebo-controlled, randomized, double-blind, cross-over clinical trial examining the effectiveness of GAA for the treatment of CFS was organized according to the CONSORT guidelines [16]. Participants who fulfilled the 1994 Centers for Disease Control and Prevention criteria for CFS [17], and who were older than 18 years of age were candidates for inclusion in the study. Exclusion criteria included: (a) psychiatric comorbidity; (b) use of any dietary supplement within four weeks prior to the study commencing; (c) unwillingness to return for follow-up analysis; and (d) pregnancy. All participants were provided informed consent to voluntarily participate in this study. The study was carried out in Serbia between 1 September 2014 and 31 May 2015. The local Institutional Review Board approved the study protocol (Ref. No. 878/13-EUTC:03). All procedures were performed in accordance with the Declaration of Helsinki. From the pool of 102 participants initially selected for the present study, 45 were checked for eligibility. Due to the fact that 80% of eligible participants were females, we exclusively recruited women into the trial to increase *sample homogeneity*. Final sample size (*n* = 21) was in accordance with the power analysis (see below) for the primary outcome measure. The mean physical characteristics of participants were: age 39.3 ± 8.8 years, weight 62.8 ± 8.5 kg, and height 169.5 ± 5.8 cm.

### 2.2. Intervention

**Figure 1:**
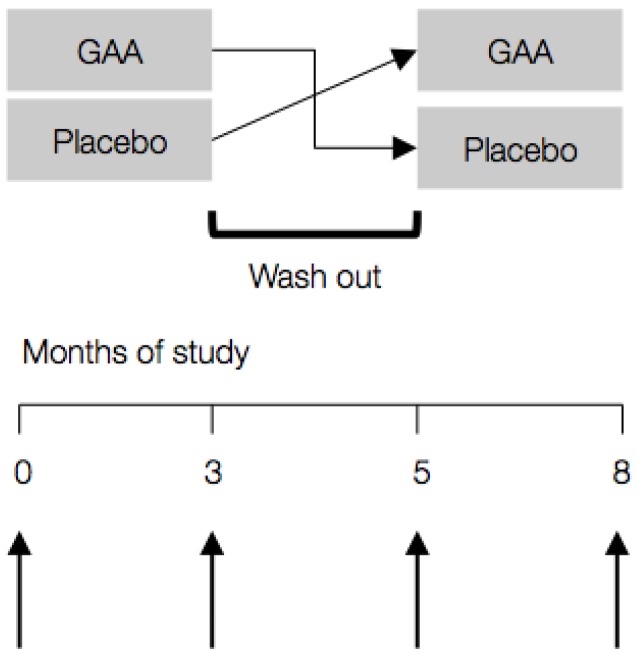
Study design with sampling intervals (↑). GAA—guanidinoacetic acid.

Participants were randomized according to a computer-generated randomization list in a double-blind design to receive either GAA (2.4 grams per day) or placebo (cellulose) by oral administration for three months; crossover was balanced with half receiving placebo first, half second (Figure 1). Wash-out period lasted for two months to prevent the residual or carry-over effects of treatments across study periods. The amount of GAA used was chosen as a dose that gives the desired effect (e.g., an increased plasma creatine concentration) with minimum side effects in men and women [15]. Participants were asked to maintain their usual lifestyle, dietary intake and not to use any dietary supplements during the study. The primary endpoint of treatment efficacy was the change in the Multidimensional Fatigue Inventory (MFI) score from baseline to three months. Additionally, assessment of health-related quality of life, exercise performance, screening laboratory studies, and side-effects evaluation were performed before and following three months of supplementation.

### 2.3. Study Protocol

At each visit to the clinic, patients were asked to complete the Multidimensional Fatigue Inventory (MFI), a 20-item questionnaire that measures global, physical and mental fatigue, and decreases in activity and motivation [18]. Each scale contains four items for which the participant had to indicate on a five-point scale to what extent the particular statement applies to her. An equal number of items was worded in either a positive or negative direction to counteract response tendencies. For each scale a total score was calculated by summing the scores of the individual items. Scores ranged from a minimum of four to the maximum of 20. A higher score is indicative of greater fatigue. Pain in the locomotive apparatus was measured with a Visual Analog Scale (VAS), in which patients separately rated their overall perceived soreness during rest and during regular physical activity, on a scale from 0 (no soreness et all) to 10 (very intensive soreness) [19]. Health-related quality of life (HRQL) was assessed through the SF-36 questionnaire [20]. The SF-36 is a self-administered questionnaire that measures HRQL according to individual self-reported health perceptions and includes two areas: physical function and emotional perception. The 36 items comprising the HRQL are grouped in eight subscales with a point range of 0–100 (in each subscale), with higher values signifying better HRQL in CFS patients [21]. The SF-36 has proven useful in surveys of general and specific populations, comparing the relative burden of diseases, and in differentiating the health benefits produced by a wide range of different treatments [22]. Test-retest reliabilities (ICC’s) for the MFI, VAS and HRQL were 0.42, 0.88 and 0.86, respectively.

Participants’ activity levels were monitored on a daily basis using actigraphy. Participant wore as actometer (ActiGraph LLC, Pensacola, FL, USA) on their waist throughout the study. Intensity level, daily duration of physical activity and active energy expenditure were recorded, and downloaded to a computer for monthly tracking, with average values calculated for the study duration. For muscular performance, maximal isometric voluntary strength of knee extensor muscles were measured bilaterally using an isometric dynamometer (Tesys 500, Globus, Codogne, Italy). Isometric strength was measured with the knee joint at 165° of flexion (180° = leg fully extended). The better of two efforts for each leg was recorded with cumulative value presented as total isometric strength. Endurance performance was assessed by an incremental walk/run tes performed on a treadmill; the participants commenced walking at a speed of 5 km·h^−1^ and speed was incremented by 1 km·h^−1^ every minute until the participant reached their maximal symptom-tolerated level. Gas-exchange data were collected throughout the test using a breath-by-breath metabolic system (Quark CPET, Cosmed, Italy). Heart rate (HR) was also recorded using a HR monitor at beat-to-beat interval (Polar S810, Kempele, Finland). All subjects were assessed on the same day with the tests performed in the same order. Patients were familiarized with exercise performance assessments during preliminary visit to the clinic two weeks before study commenced.

For biochemical analyses, patients provided both fasting blood samples and 24-h urine samples at each visit to the clinic. Serum and urinary GAA, creatine and creatinine were measured by HPLC with fluorimetric detection (Hewlett-Packard, Palo Alto, CA, USA). Total serum homocysteine was determined with chemiluminescent immuno-assay method using chemistry analyzer (DPC Immulite 2000, Siemens, Germany). A complete blood count was performed using a Coulter blood counter (Model S-plus II, Coulter Electronics Inc., Hialeah, FL, USA) and yielded values for red blood cell count (RBC), white blood cell count, platelets, hemoglobin, hematocrit, and RBC indices. Erythrocyte sedimentation rate (ESR) was measured by the Westergren method. Glucose, total cholesterol, triglycerides, and lipoprotein levels were analyzed by standard enzymatic methods with automated analyzer (Hitachi 704, Tokyo, Japan). Serum *sodium*, *potassium* and *calcium levels were analyzed* by ISE direct with ILyte analyzer (Kaunas, Lithuania). Serum activities of aspartate transaminase, alanine transaminase, *lactate dehydrogenase*, *alkaline phosphatase*, and creatine kinase were *analyzed* by an automated analyzer (RX *Daytona, Randox* Laboratories Ltd., Crumlin, UK). Urine protein, blood and glucose were analyzed by standard screening test (Machery-Nagel GmbH & Co. KG, Duren, Germany). All samples for each subject were assayed in the same run. For all values, the first reading was discarded and the mean of the next three consecutive readings with a coefficient of variation below 15% were used in the study. In addition, resting skeletal muscle creatine concentration was determined non-invasively by 1.5 Tesla proton magnetic resonance (MR) spectroscopy (Signa, General Electric, Fairfield, CT, USA). MR spectra were collected with dual radio frequency transmit-receive surface coil (10 cm) placed over the vastus lateralis muscle of the dominant leg in patients lying in a supine position for 15 min. The repetition time was 2.5 s, with the free induction decay sampling rate 2000 Hz, and 1024 sampling points and averages. Relative concentrations of creatine in mmol·kg^−1^ wet weight were calculated as relative concentrations related to the total peak area of the spectrum [23]. In addition, participants were instructed to report on adverse effects of intervention through open-ended questionnaire at the end of intervention.

### 2.4. Statistical Analyses

The primary efficacy outcome was the change in MFI score (general fatigue) at three months after administration (effect size of 1.0) in the GAA group over the placebo group. Allowing for 90% power and alpha level set to 0.05, it was estimated that 13 participants would be required in the final analyses (G*Power 3, Heinrich-Heine-Universität Düsseldorf, Germany). This was adjusted to 21 subjects to account for a predicted 30% dropout. When homogenous variances were verified for normally distributed data, measures were compared by two-way mixed model ANOVA with repeated measures to establish if any significant differences existed between participants’ responses over time of intervention (baseline *vs.* post-administration), with the intervention (GAA or placebo) included as between-subjects factor. When non-homogenous variances were identified, values were compared using Kruskal-Wallis test. The analyses were performed in the modified intention-to-treat population. Significance level was set at *p* ≤ 0.05. All results were expressed as mean ± standard deviation (SD).

## 3. Results

A total of 14 participants completed the follow-up measures. Seven participants were lost during the intervention period due to reasons not connected to the study *per se.* No single participant was excluded during the study due to adverse events. Changes in MFI-20, HRQL and musculoskeletal pain during the study (baseline *vs.* post-administration at three months) are presented in Table 1. No effects of the intervention were found for the primary efficacy outcome (MFI score for general fatigue) or physical fatigue. GAA attenuated other aspects of fatigue (higher scores indicate a higher degree of fatigue) such as activity, motivation and mental fatigue (*p* < 0.05). In addition, results indicated no significant treatment *vs.* time interaction for musculoskeletal soreness at rest and during regular physical activity, while a significant interaction was found for health-related quality of life outcomes (*p* < 0.05). GAA treatment improved both physical and mental common scores at post-administration compared to placebo administration (*p* < 0.05).

**Table 1:** Changes in multidimensional fatigue scores, health-related quality of life, and musculoskeletal soreness from baseline to three months. Values are mean ± SD. GAA- guanidinoacetic acid.

	Baseline	At Follow up		p *
		Placebo	GAA	
**Multidimensional fatigue score**				
General fatigue	12.1 ± 1.5	11.8 ± 1.5	11.6 ± 1.3	0.44
Physical fatigue	11.2 ± 1.0	11.6 ± 1.4	11.7 ± 1.2	0.99
Reduced activity	11.7 ± 1.6	13.9 ± 1.2	11.7 ± 1.8	0.00
Reduced motivation	15.2 ± 1.5	15.0 ± 1.8	13.1 ± 1.9	0.03
Mental fatigue	12.9 ± 1.3	14.0 ± 0.9	12.2 ± 1.7	0.01
**Musculoskeletal soreness**				
At rest (score)	1.4 ± 1.1	1.4 ± 1.3	1.2 ± 1.0	0.31
During activity (score)	5.0 ± 1.5	5.0 ± 1.8	4.4 ± 1.5	0.18
**Health-related quality of life**				
Physical common score	55.1 ± 4.9	52.8 ± 4.2	55.2 ± 2.8	0.04
Mental common score	42.4 ± 13.3	45.8 ± 6.5	51.1 ± 5.5	0.00

*Note* * *p* value from two-way ANOVA with repeated measures for treatment *vs.* time interaction.

**Figure 2:**
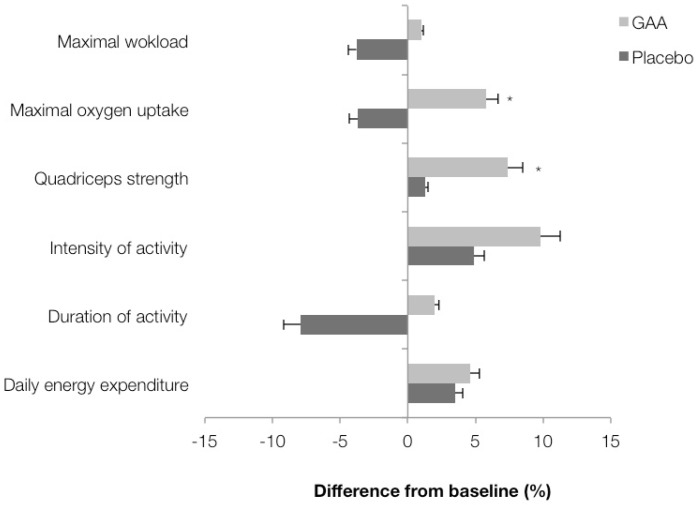
Percentage change in exercise performance end points zero *vs.* three months. Values are mean ± SD. Asterisk (*) indicates significant interaction effect (treatment *vs.* time) at *p* < 0.05.

Changes in exercise performance from baseline to three months post-administration are presented in Figure 2. Significant differences in the percent change in total quadriceps isometric strength and maximal oxygen uptake were observed between the interventions (*p* < 0.05), while no differences were noted for daily energy expenditure (*p* = 0.98), physical activity duration (*p* = 0.23) and intensity (*p* = 0.22). A trend (*p* = 0.08) towards a difference was noted in maximal workload during ergometry between the GAA and placebo groups.

Levels of serum and urinary guanidino compounds at baseline and at post-administration are presented in Table 2. GAA intervention significantly affected all guanidino compounds (*p* < 0.05) except for urinary creatine, as compared to the placebo. In addition, after three months of intervention, participants receiving GAA significantly improved muscular creatine concentrations compared with the placebo (36.3% *vs.* 2.4%; *p* < 0.01). Intervention had no effect on blood glucose and lipid profiles, liver and muscle enzymes, hematological indices and urinary outcomes (not presented).

**Table 2:** Changes in serum and urinary guanidines, and muscle metabolites during the study. Values are mean ± SD.

	Baseline	At Follow up		p *
		Placebo	GAA	
**Serum**				
GAA (µmol/L)	3.0 ± 0.3	2.6 ± 0.4	4.2 ± 1.2	<0.001
Creatine (µmol/L)	26.1 ± 5.1	35.3 ± 12.8	47.8 ± 13.5	0.048
Creatinine (µmol/L)	77.1 ± 7.8	77.7 ± 13.0	100.7 ± 14.6	<0.001
Homocysteine (µmol/L)	9.6 ± 1.7	9.4 ± 1.8	11.9 ± 1.6	<0.001
**Urine**				
GAA (µmol/L)	151.9 ± 45.2	146.9 ± 50.6	274.1 ± 101.1	0.004
Creatine (µmol/L)	17.6 ± 2.3	29.3 ± 11.2	42.8 ± 22.7	0.192
Creatinine (µmol/L)	1.0 ± 0.3	1.3 ± 0.4	1.7 ± 0.4	0.041
**Muscle**				
Creatine (mmol/kg wet weight)	27.8 ± 4.5	28.5 ± 4.8	38.0 ± 2.6	0.008

*Note:* * *p* value from two-way ANOVA with repeated measures for treatment *vs.* time interaction.

## 4. Discussion

The results of the present study indicated that general fatigue and musculoskeletal soreness were not affected by three months of GAA supplementation in adult women with chronic fatigue syndrome (CFS). On the other hand, participants that consumed GAA appeared to have significantly greater motivation and improved health-related quality of life measures. In addition, daily ingestion of 2.4 grams of GAA for three months improved muscular strength and aerobic power, while other aspects of exercise performance were not affected by the intervention. GAA intake appears to affect the metabolism of creatine, with a significant increase in fasting serum creatine and skeletal muscle creatine levels at post-administration, accompanied with elevated serum homocysteine in participants receiving GAA.

CFS is a debilitating and complex illness of unknown etiology, also known as fibromyalgia, myalgic encephalomyelitis or systemic exertion intolerance disease [7]. Characterized by persistent disabling fatigue that cannot be explained by other conditions, CFS results in a wide range of presenting symptoms such as a substantial reduction in activity level, multi-site muscle and joint pain, or general malaise, with women appearing to be more affected than men [24]. Numerous factors including previous psychiatric disorder, stressful events, high academic achievement, infections and others have been suggested to have a role in contributing to the etiology of CFS, but there is little evidence to implicate any of these as risk factors [25,26]. Treatment options include cognitive behavioral therapy, graded exercise therapy, nutritional intervention and pharmacological therapy, yet no pharmacological or complementary therapy has been proven to be sufficiently effective [27,28]. Since patients with CFS show decreased resting values of phosphocreatine in the skeletal muscle [3], restoration of muscle creatine stores might positively affect cellular bioenergetics, and improve fatigue levels and clinical symptomatology. GAA acts as a highly bioavailable creatine precursor [15], and previous research reported energy-boosting effects of GAA administration in the clinical environment [29]. In this study, we found that oral administration of GAA for three months increased creatine levels in the skeletal muscle in women with CFS, yet no effects were reported for main clinical outcomes, such as general fatigue and musculoskeletal soreness, or maximal workload during exercise. Furthermore, GAA intervention was ineffective in increasing daily duration of physical activity and active energy expenditure, as assessed by actigraphy. It seems that some dietary GAA is metabolized to yield creatine, resulting in a significant increase in serum and muscle creatine after intervention, which is in accordance with previous GAA studies in humans [15]. However, the sense of fatigue and pain, and reduced activity, as prominent disabling features seem to poorly associate with creatine availability in women with CFS. On the other hand, GAA improved other patient-reported outcomes, such as health-related quality of life, mental fatigue or motivation, which to some extent justify the use of GAA in this clinical population. In addition, GAA improved lower-body muscular strength and cardiorespiratory endurance. Therefore, supplemental GAA might be of true benefit for the population with CFS to cope with everyday physical activities. Performance-enhancing effects of GAA are probably due to the “creatine recovery” effect of GAA [30], yet GAA may elicit improvements in exercise performance through different means, which requires further investigation. As a more stable, bioavailable and cost-effective replacement for creatine, dietary GAA might positively affect protein utilization, neuromodulation, and oxidant-antioxidant status in clinical population [31]. On the other hand, creatine supplementation might provide the same energy-boosting benefits as GAA while avoiding the increase in homocysteine. Therefore, studies comparing GAA and creatine in the clinical environment, including in patients with CFS, are warranted.

No side effects were reported from the participants in this present study. This is consistent with previous research that has reported that GAA supplementation is associated with a low incidence of biochemical abnormalities in healthy men and women [15]. However, GAA intervention increased serum T-Hcy by approximately 2.3 µmol/L (24%) in participants at post-administration. Since abnormally high levels of T-Hcy in serum (above 15 µmol/L) is an independent risk factor for cardiovascular and neurodegenerative diseases [32], an increase in plasma T-Hcy driven by dietary GAA might be clinically relevant. In the present study serum T-Hcy remained below the clinically significant level for all participants during the intervention, suggesting low risk of hyperhomocysteinema in female patients supplemented with 2.4 g/day of GAA for three months. However, more studies are warranted to further evaluate long-term safety and effectiveness of GAA supplementation in the clinical environment, particularly in patients at cardiovascular risk and/or with disturbances in homocysteine metabolism.

## 5. Conclusions

In conclusion, we found no significant effects of three months of GAA administration (2.4 grams daily) on general fatigue and muscle soreness in women with CFS. However, GAA improved health-related quality of life measures and reduced mental fatigue. GAA also ameliorated muscular strength and maximal oxygen uptake in CFS patients, which was likely related to the elevations in muscle phosphocreatine levels. Supplemental GAA had an acceptable safety profile, with no evidence of hyperhomocysteinemia in adult women with CFS.

## Abbreviations

CFS: Chronic fatigue syndrome
ESR: Erythrocyte sedimentation rate
GAA: Guanidinoacetic acid
HR: Heart rate
HRQL: Health-related quality of life
MFI: Multidimensional fatigue inventory
MR: magnetic resonance
RBC: Red blood cell count
VAS: Visual analog scale
